# The diverse roles of glutathione-associated cell resistance against hypericin photodynamic therapy

**DOI:** 10.1016/j.redox.2017.02.018

**Published:** 2017-02-24

**Authors:** Theodossis A. Theodossiou, Cathrine E. Olsen, Marte Jonsson, Andreas Kubin, John S. Hothersall, Kristian Berg

**Affiliations:** a Department of Radiation Biology, Institute for cancer Research, Radium Hospital, Oslo University Hospital, Montebello, 0379 Oslo, Norway; b PLANTA Naturstoffe Vertriebs GmbH, A-1120 Wien, Austria

**Keywords:** Photodynamic therapy of Cancer, Hypericin, Glutathione, Predictive markers for PDT, Glutathione peroxidases, Glutathione-S- transferases

## Abstract

The diverse responses of different cancers to treatments such as photodynamic therapy of cancer (PDT) have fueled a growing need for reliable predictive markers for treatment outcome. In the present work we have studied the differential response of two phenotypically and genotypically different breast adenocarcinoma cell lines, MCF7 and MDA-MB-231, to hypericin PDT (HYP-PDT). MDA-MB-231 cells were 70% more sensitive to HYP PDT than MCF7 cells at LD_50_. MCF7 were found to express a substantially higher level of glutathione peroxidase (GPX4) than MDA-MB-231, while MDA-MB-231 differentially expressed glutathione-S-transferase (GSTP1), mainly used for xenobiotic detoxification. Eighty % reduction of intracellular glutathione (GSH) by buthionine sulfoximine (BSO), largely enhanced the sensitivity of the GSTP1 expressing MDA-MB-231 cells to HYP-PDT, but not in MCF7 cells. Further inhibition of the GSH reduction however by carmustine (BCNU) resulted in an enhanced sensitivity of MCF7 to HYP-PDT. HYP loading studies suggested that HYP can be a substrate of GSTP for GSH conjugation as BSO enhanced the cellular HYP accumulation by 20% in MDA-MB-231 cells, but not in MCF7 cells. Studies in solutions showed that L-cysteine can bind the GSTP substrate CDNB in the absence of GSTP. This means that the GSTP-lacking MCF7 may use L-cysteine for xenobiotic detoxification, especially during GSH synthesis inhibition, which leads to L-cysteine build-up. This was confirmed by the lowered accumulation of HYP in both cell lines in the presence of BSO and the L-cysteine source NAC. NAC reduced the sensitivity of MCF7, but not MDA-MB-231, cells to HYP PDT which is in accordance with the antioxidant effects of L-cysteine and its potential as a GSTP substrate. As a conclusion we have herein shown that the different GSH based cell defense mechanisms can be utilized as predictive markers for the outcome of PDT and as a guide for selecting optimal combination strategies.

## Highlights

• The intracellular use of glutathione is proposed as a predictive marker for PDT.
• Cells with higher levels of GPX enzymes are more resistant to PDT.
• Cells with higher levels of GSTP1 can detoxify xenobiotics and also photosensitizers.
• L-cysteine can bind GSTP1 substrate CDNB equally in the absence/presence of GSTP1.
• L-cysteine can bind CDNB as efficiently as glutathione in the presence of GSTP1.

## Graphical abstract

**Figure:**
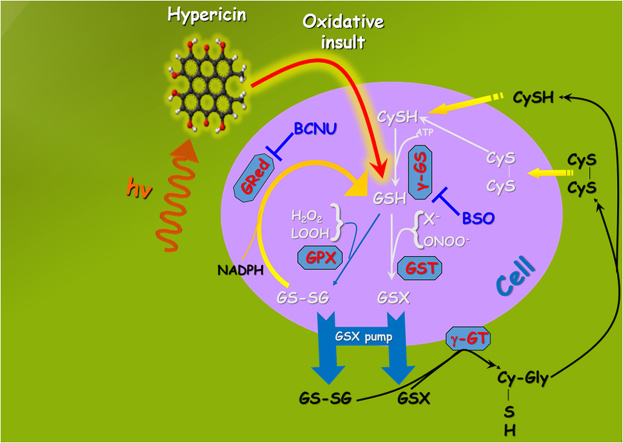

## 1 Introduction

Photodynamic Therapy is a photomedical treatment using a photosensitive substance, photosensitizer (PS), which upon irradiation by light at the appropriate wavelength, interacts with biomolecules or molecular oxygen to produce reactive species. PDT has been approved for several indications, in particular for cancerous diseases, but mainly as a palliative end point. Despite that PDT has been evaluated in clinical trials for more than 30 years and despite approvals for clinical use, PDT has not so far become part of standard clinical practice. In recent years molecular biology-based research, including deep sequencing analyses, has documented the large tumor heterogeneity even between tumors of the same origin and sub-classification [1]. This has led to development of personalized medicine based on predictive markers for treatment response and is expected to result in the use of approved drugs and treatment modalities on only a subfraction of tumors today treated uniformly [2]. This has already shown to be a good strategy for treating EGFR positive colon cancer depending on their ras status [3]. The differences in tumor sensitivity to PDT have been shown to be large although little has been done to reveal the mechanistic basis for this difference [4]. Studies of mechanisms influencing the cell sensitivity to PDT may be utilized in the search for predictive markers for PDT response and better selection of patients to undergo PDT. In this way the clinical benefit of PDT may be more easily documented and lead to better selection of patients and therapeutic regime.

PDT is under consideration for treatment of breast cancers [5]. However, molecular expression profiles have shown that breast cancers may be divided in several subgroups with different sensitivity to various treatments [6] that may also include PDT. In addition, standard therapies include ionizing radiation, various chemotherapeutic and hormone based therapies as well as mAbs such as Herceptin. These treatment modalities induce resistance mechanisms that could influence second line treatments such as PDT. Thus, it is of outermost importance to reveal the predictive markers for PDT sensitivity of the cancer cells and in this way select eligible patients for PDT.

In order to seek knowledge regarding possible predictive markers for sensitivity to PDT, two invasive ductal/breast carcinoma cell lines (MCF7 and MDA MB 231) have been selected in this study, for the following reasons: (i) MCF7 cells are estrogen and progesterone receptor positive, while MDA-MB-231cells are triple negative; (ii) MCF7 cells prefer oxidative phosphorylation for ATP production at normoxic conditions (Pasteur type metabolism) and switch to glycolysis under hypoxia, whilst MDA-MB-231 cells rely on glycolysis for ATP production in both normoxic and hypoxic circumstances (Warburg type metabolism) [7] and (iii) MCF7 cells express the epithelial phenotype while MDA-MB-231 cells express mesenchymal characteristics [8]. Also, MDA-MB-231 breast cancer cells have been documented for their multidrug resistance [9], and this has been attributed to e.g P-gp upregulation [9]. The lack of estrogen receptors (ER) has rendered MDA-MB-231 cells insensitive to treatments with antiestrogens, such as tamoxifen [10], which is widely used in breast cancer chemoprevention [11], but also as an adjuvant in treatment of primary disease [12].

In the present project, we endeavoured to mechanistically elucidate the different responses to hypericin (HYP)-PDT between MCF7 and MDA-MB-231 cells, which we observed in the course of experiments, based on their relevant differences. It has to be noted that the recorded difference in photocytotoxicity between the two cell lines was also detected previously, in our recent work on bimodal porphyrin-cyclodextrin system [13]. In that work the phototoxicity was conferred by 5,10,15,20-Tetrakis(*m*-hydroxyphenyl)-21,23*H*-porphyrin. A profound discrepancy between the responses of the two cell lines to TPCS_2a_ PDT was also observed elsewhere [14]. In that study MCF7 cells were practically insensitive to TPCS_2a_ PDT, while MDA-MB-231 were very efficiently decimated by the same treatment.

## 2 Materials and methods

*Chemicals and reagents* RPMI 1640 without phenol red, L-Glutamine, penicillin/streptomycin, trypsin, dimethylsulfoxide (DMSO), Antimycin A (ANTI-A), *N*-Acetyl-L-cysteine (NAC), Ethylenediaminetetraacetic acid (EDTA), trizma® hydrochloride (TRIS-HCL), trizma® base (TRIS-BASE), , polyethylene glycol sorbitan monolaurate (TWEEN 20), bovine serum albumin (BSA), Buthionine sulfoximine (BSO), Carmustine (BCNU), Triton X-100, thiazolyl blue tetrazolium bromide (MTT), β-Nicotinamide adenine dinucleotide reduced disodium salt (NADH), metaphosphoric acid (MPA), triethanolamnine (TEAM), anti-γ-tubulin, Glutathione S-Transferase (GST) Assay Kit and sodium pyruvate were purchased from Sigma-Aldrich Norway AS (Oslo, Norway), Anti-GPX-4 (H-90) from Santa Cruz Biotechnology, Inc. (Santa Cruz, CA, U.S.A.) and anti-GSTP1 (3F2) from Cell Signalling Technology, Inc. (Danvers, MA, U.S.A.). Hypericin (HYP, 99.3%) was obtained from Planta Natural Products GmbH (Vienna, Austria). The total glutathione (GSH) assay kit and 17β-Estradiol (E2) were purchased from Cayman Chemical Company (Ann Arbor, MI, USA).

### 2.1 Cell culture

The MCF7 and MDA-MB-231 (triple negative) human breast adenocarcinoma cell lines were purchased from ATCC. Both cell lines were grown in RPMI 1640 media without phenol red, supplemented with 10% FBS, 100 U/mL penicillin/100 μg/mL streptomycin and 2 mM L-Glutamine at 37 °C in a 5% CO_2_, humidified atmosphere. Cells were inoculated into 96-well plates (20×10^3^ cells/100 μL media/well) or 6 well plates (1×10^6^ cells/2 mL media/ well), 24 h prior to treatment.

### 2.2 Cell treatment

BSO was added to cells overnight and was subsequently maintained in the appropriate treatment groups until cytotoxicity assessment. NAC was added to the appropriate cell groups together with HYP (4 h before irradiation) and maintained up to the cytotoxicity assessment (24 h after irradiation). The DMSO content (where applicable) was at all times kept ≤0.25%. Following cell incubation with HYP for 4 h, all treatment groups were washed twice. The cells were irradiated from the plate underside by means of a *Lumisource* lamp (PCI Biotech AS, Oslo, Norway) through a 530 nm cut-off longpass filter (Roscolab Ltd, London, U.K.), at an irradiance of 4 mW/cm^2^.

### 2.3 Cytotoxicity assessment

Cells were inoculated (20×10^3^) into 96-well plates and left to incubate in complete media containing 10% FBS for 24 h. Cells were then treated with media only or HYP (2 μM), in the presence of modulators (where appropriate) and irradiated. The cell viability was assessed by the MTT assay 24 h post-irradiation. The assay was performed by replacing cell media with complete media containing 0.5 mg/mL MTT and incubating at 37 °C in a 5% CO_2_ humidified atmosphere for 3 h. MTT media were subsequently aspirated from all cells and the produced formazan crystals solubilized with 100 μL DMSO per well. The plates were shaken for 10 min at ~300 rpm in a Heidolph Titramax 101 orbital shaker (Heidolph Instruments GmbH & Co.KG), and the endpoint absorbance measurements at 570 nm were performed in a BioTek PowerWave XS2 plate reader (BioTek Instruments, Inc.). Blank values measured in wells with DMSO and no cells, were in all cases subtracted.

### 2.4 Hypericin loading

Fluorescence spectroscopy was employed to monitor HYP loading in MCF7 and MDA-MB-231 cells. Cells were seeded in 96 well plates. The designated groups were treated with 100 μM BSO overnight. Subsequently cells were treated with vehicle, HYP, BSO+HYP, NAC+HYP and BSO+NAC+HYP for 4 h. All cells were consequently washed twice and all cell groups were placed in complete media. The fluorescence was read in a Biotek synergy 2 platereader (BioTek Instruments, Inc.) using a 530±25 nm bandpass excitation filter and a 590±35 nm bandpass emission filter. Empty wells incubated with HYP and washed twice were used as blanks and subtracted from all data groups.

### 2.5 GSH measurements

The total GSH was measured according to the Tietze recycling assay [15], following the manufacturers’ (Cayman Chemical) instructions. Cells seeded in 96 well plates (20×10^3^ per well) were left overnight to attached and then the designated cell groups were treated with BSO (again overnight). Next, the cells were treated with HYP for 4 h, in the presence and absence of BSO and NAC and then irradiated. The corresponding dark controls were included. Irradiated samples were assayed immediately and 2 h post-irradiation. In all samples 200 μL of 0.5 w/v metaphosphoric acid (MPA) was added to the cells for protein precipitation together with 1 mM EDTA to prevent GSH oxidation from transition metals at the desired assay point, and the cells were kept at −20 °C until the GSH assay was performed. 3 μL of 4 M triethanolamine (TEAM) was added to each well to neutralize the MPA prior to the assay. Fifty microliters of each well supernatant was assayed for GSH content for all treatment groups.

### 2.6 Conjugation of GSH, NAC and L-cysteine to 1-Chloro-2,4-dinitrobenzene (CDNB) in the presence and absence of GST

In order to investigate the ability of NAC to bind the GST substrate CDNB in the presence and absence of the catalytic enzyme glutathione S transferase, in comparison to GSH and L-cysteine we devised the following assay: In a 1 cm path length (1 mL) quartz cuvette we added 980 μL milli-Q water, 10 μL of 200 mM GSH, NAC or L-cyst, 10 μL of 100 mM CDNB in the presence and absence of 4 μL (1 μg) GST. The rate of conjugation was monitored via absorbance measurements at 340 nm with the use of a UV-2550 UV–VIS spectrophotometer (Shimatdzu, Kyoto, Japan), during 6 min, at 0.5 min intervals. The conjugation rates were calculated as $\mathrm{CR}=\frac{{\Delta Α}_{340\mathrm{nm}}/m\mathrm{in}}{\varepsilon_{\mathrm{mM}}}$, where ε_mM_=9.6 mM^−1^ cm^−1^.

### 2.7 Western blots

Cellsgrown in 6 well plates were harvested (~10^6^) at 6 h following irradiation of groups treated with vehicle, 4-OHT (post-irradiation, 15 μM, 4 h), HYP (2 μM, 4 h) or 4-OHT (post-irradiation) + HYP. The lysates were sonicated in a 4710 series Cole-Palmer ultrasonic homogenizer (100% duty cycle) and boiled for 5 min at 95 °C. Appropriate amounts of lysates (15 μL maximum) were loaded onto Criterion™ TGX™ precast gels (Bio-Rad Laboratories Inc.) and ran at 200 V on ice. The proteins were subsequently transferred from the gels onto nitrocellulose membranes, using a Trans-Blot® Turbo™ transfer system (Bio-Rad Laboratories Inc.). The membranes were subsequently washed with TTBS and blocked with 5 w/v skimmed milk for 1 h at RT. The membranes were then incubated overnight at 4 °C with the selected primary antibodies, diluted according to their manufacturers’ recommendations in 5 w/v skimmed milk. The membranes were then washed three times in Tween-Tris-buffered Saline (TTBS, 5 min per wash) and incubated with the secondary antibodies according to the suppliers’ indications. The membranes were again washed three times with TTBS and incubated for 5 min with LumiGlo (KPL, Kirkegaard & Perry Laboratories, Inc.). The western blots were read at ChemiDoc™ MP Imaging System (Bio-Rad Laboratories Inc.); γ-tubulin was in all cases used as a loading control.

## 3 Results

**Fig. 1:**
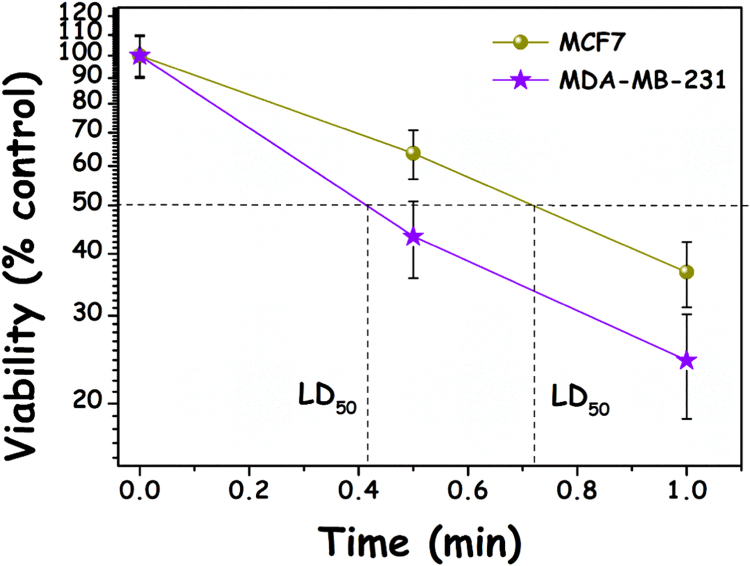
HYP-PDT light dose response in MCF7 cells (dark yellow spheres) and MDA-MB-231 (blue stars) breast ductal carcinoma cells. Both cell lines were incubated with 2 μM HYP for 4 h, and washed twice prior to irradiation. The irradiance was measured as 4 mW/cm^2^. Cell viability was assessed 24 h post-irradiation by standard MTT assays. (For interpretation of the references to color in this figure legend, the reader is referred to the web version of this article.).

**Fig. 2:**
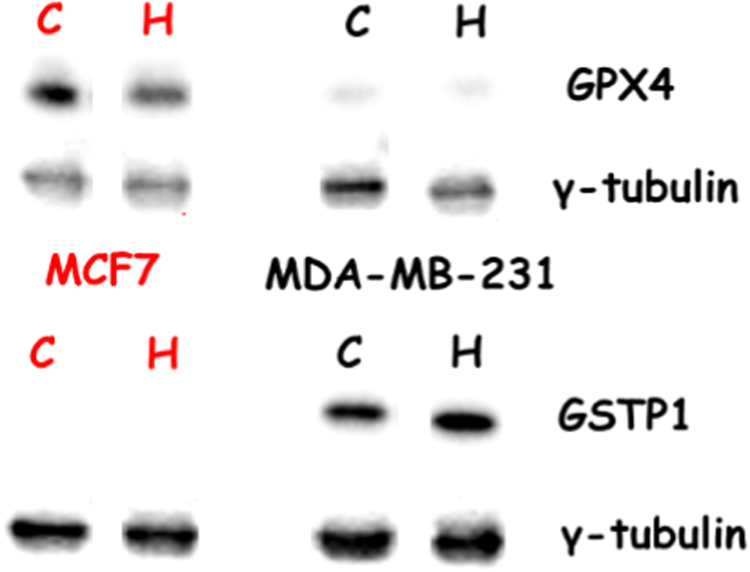
Western blots for GPX4 (top set) and GSTP1 (bottom set) in MCF7 (annotation in red, left) and MDA-MB-231 (annotation in black, right) cells. The blots were performed in media only control cells (C) and HYP-PDT treated cells (H). All lysates were harvested 6 h post-irradiation. The house-keeping gene of choice was γ-tubulin. (For interpretation of the references to color in this figure legend, the reader is referred to the web version of this article.).

In a course of PDT experiments with HYP on MCF7 and MDA-MB-231 cells, we found that MCF7 cells were more resilient to PDT than MDA-MB-231 cells (Fig. 1). In four separate experiments where the cells were exposed to 45 s of light, respectively 47±10% and 27±5% of the MCF7 and MDA-MB-231 cells survived (p=0,019, two-tailed *t*-test). In our effort to find the cause behind this difference in PDT sensitivity, we have chosen to evaluate the impact of the cellular glutathione as a defence mechanism against ROS formation. In this respect MCF7 but not MDA-MB-231 cells were found to express the membrane resident antioxidant protein glutathione peroxidase 4 (GPX-4), which detoxifies lipid hydroperoxides (LOOHs). It was also found that HYP PDT reduced the level of GPX-4 in MCF7 cells and this is possibly attributed to the photodynamic inactivation of the membranic protein (or destruction of the antibody binding site), especially since HYP is a very lipophilic photosensitizer (Fig. 2). In contrast, MDA-MB-231 cells, but not MCF7 cells, were found to express glutathione S transferase p1 (GSTP1), a member of the GST family of enzymes (Fig. 2). Soluble GSTs are divided into 4 main categories: alpha, mu, pi, and theta. GSTP1 proteins function in xenobiotic metabolism by conjugating many hydrophobic and electrophilic compounds with GSH, so that the conjugates can be subsequently expelled from the cells by the GS-X pump [16]. These findings led us to assume that whilst MDA-MB-231 cells are more susceptible to HYP phototoxicity due to their lack of the membranic GPX-4 antioxidant defences, they probably utilize glutathione (GSH) to bind to xenobiotics and/or photooxidized molecules and expel them with the help of GSTP1.

**Fig. 3:**
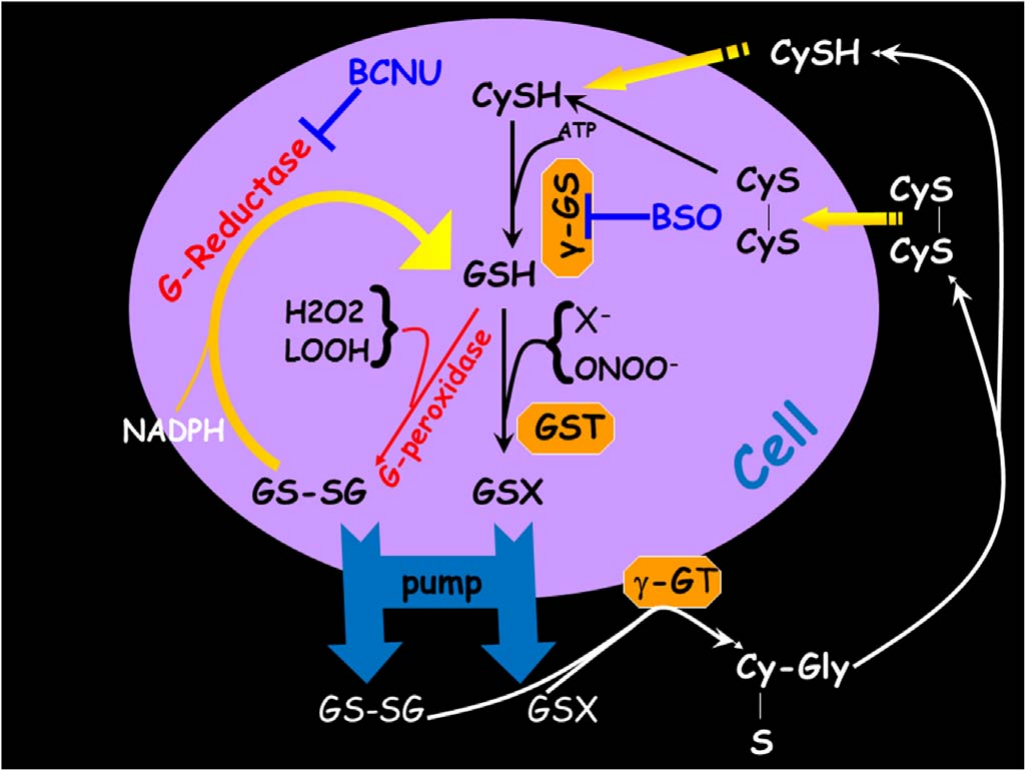
Intracellular glutathione biochemistry.

**Fig. 4:**
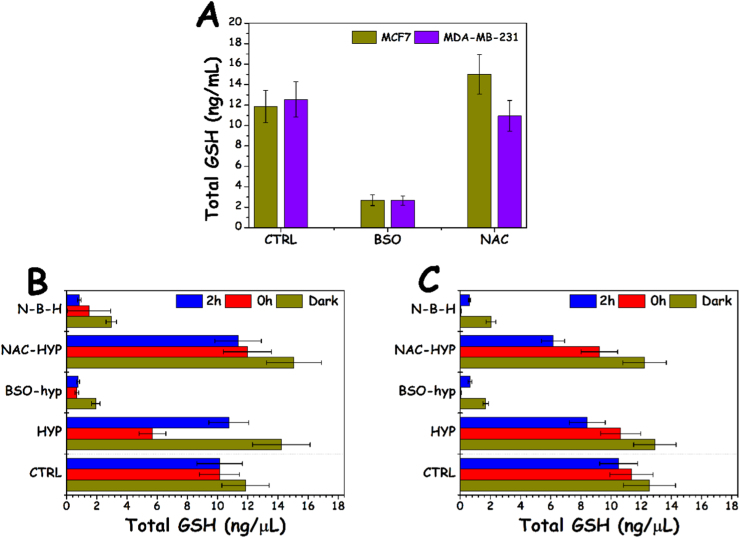
Total GSH measurement: A. MCF7 and MDA-MB-231 cells treated overnight with 100 μM BSO and with 3 mM NAC for 4 h, B. MCF7 cells treated with HYP±light (2 μM, 4 h) and BSO (100 μM, overnight) and/or NAC (3 mM, 4 h) and C. MDA-MB-231 cells as in B. In B,C GSH was measured at two time points, 0 and 2 h following irradiation of the HYP-PDT groups, and also for dark controls.

**Fig. 5:**
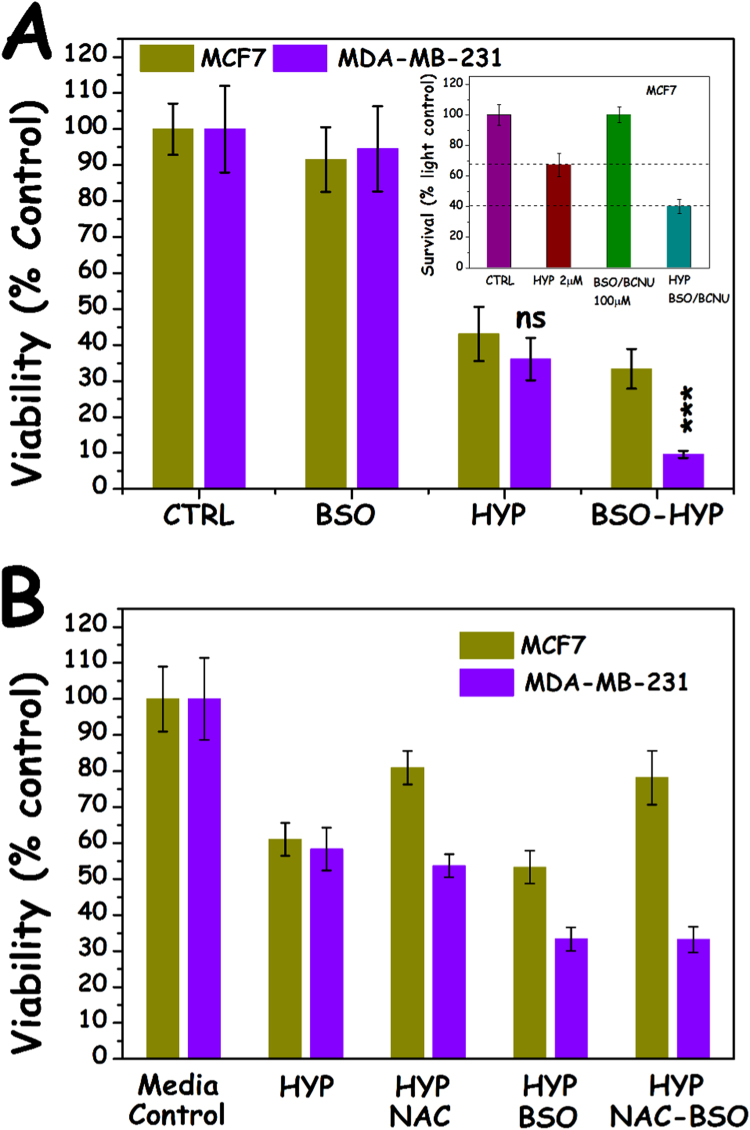
The effect of GSH on cell viability following HYP-PDT. A) MCF7 (yellow bars, left) and MDA-MB-231 (blue-bars, right), with and without overnight pretreatment wit 100 μM BSO (inhibitor of glutathione synthetase) and B) as A but also in the presence/absence of the (non γ-glutamyl) cysteine antioxidant NAC (3 mM). Inset: MCF7 cells pretreated with both BSO and BCNU (Inhibitor of glutathione reductase 100 μM). The light doses employed were 200 mJ cm^−2^ for MCF7 and 100 mJ cm^−2^ for MDA-MB-231 cells. (For interpretation of the references to color in this figure legend, the reader is referred to the web version of this article.).

In order to further investigate the differential use of GSH in the two cell lines we employed overnight pretreatment with BSO (an inhibitor of de*-novo* intracellular GSH synthesis Fig. 3), prior to HYP PDT. Overnight treatment with 100 μM BSO was found to deplete intracellular GSH by ~80%, in both cell lines (Fig. 4A), without notably compromising their viability in the absence of an oxidative insult. From the results in Fig. 5A it appears that treatment of MCF7 cells with BSO does not have a statistically significant effect on HYP PDT cytotoxicity while in MDA-MB-231 cells GSH depletion causes a profound exacerbation of HYP PDT cell death (p=0.013 two tailed *t*-test, n=4). In an attempt to further reduce the intracellular GSH levels, MCF7 cells were treated with both BSO and BCNU (GSH reductase inhibitor, 100 μM, overnight, Fig. 5A insert). The pretreatment of MCF7 cells with BSO and BCNU significantly enhanced the cytotoxicity of HYP PDT. This indicates that 80% reduction of GSH by BSO was not enough to confer differential cell death and the remaining GSH from GS-SG reduction (*de salvo*) was enough to defend against HYP PDT enzymatically via GPX4. In parallel experiments we consequently used NAC (3 mM), a cell permeable cysteine antioxidant, in BSO treated and untreated cells. From the results shown in Fig. 5B, NAC seemed to significantly protect MCF7 cells from HYP PDT in the present and absence of BSO, while it was without any effect in the case of MDA-MB-231 cells.

In order to further understand the impact of NAC, BSO and HYP PDT on the intracellular content of GSH and cell survival the total GSH for the various treatment groups was analysed (Fig. 4). As stated earlier, in both cell lines overnight pretreatment with BSO depleted the GSH content to ~20% of controls. Addition of NAC for 4 h led to a minor increase of GSH by +20% in otherwise untreated MCF7 cells (p=0.025), but not in MDA-MB-231 cells (Fig. 4A). The application of HYP PDT on MCF7 cells substantially depleted the GSH content by about 50% immediately following light exposure, whilst 2 h later the GSH level recovered to 80% of the control value. In the case of MDA-MB-231 cells, however, the levels of GSH following HYP PDT only dropped by ~20% and by two hours it had merely further dropped to two thirds of control.

**Fig. 6:**
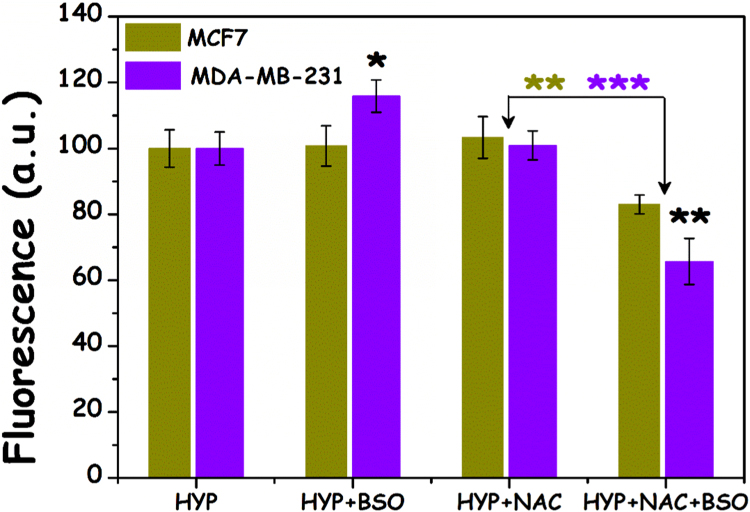
HYP (2 μM, 4 h) loading±BSO (100 μM, overnight pretreatment) and ±NAC (3 mM, 4 h) in MCF7 and MDA-MB-231 cells. The loading was measured by fluorescence (λex=530±25 nm, λem=590±35 nm) after the 4 h incubation.

HYP may be a substrate for GSTP conjugation to GSH and thereby excreted from the cells resulting in lower HYP levels in the cells and reduced sensitivity in PDT. We studied the cellular accumulation of HYP in both MCF7 and MDA-MB-231 cell lines (Fig. 6). MDA-MB-231 cells were found to exhibit a slightly higher HYP uptake when pretreated with BSO overnight, most probably due to reduced GST-mediated HYP expulsion from the cell, following GSH depletion. Surprisingly, however, both cell lines exhibited a profound reduction in HYP uptake following pretreatment with both BSO and NAC, but not in the case of pretreatment just with NAC. This reduction was larger for MDA-MB-231 cells, and could be accounted to their GST expression, however it was puzzling in the case of MCF7 which do not express GST (*vide supra*).

In order to resolve this anomaly, we performed conjugation experiments between GSH, NAC and L-cyst with the GST substrate CDNB in the presence (enzymatically) and absence (spontaneously) of GST. From these rates (Table 1) it is obvious that GSH has a low spontaneous rate of conjugation which is profoundly increased in the presence of GST. This is expected as GSH mainly binds xenobiotics enzymatically for subsequent expulsion from the cell. In the case of NAC however both the spontaneous and enzymatic binding rates were quite small indicating no obvious significant interaction of NAC with xenobiotics in the presence or absence of GST. In cells NAC is hydrolized enzymatically or spontaneously into L-cysteine [17] which is the precursor of GSH. In this context we were intrigued to also test L-cyst for its xenobiotic binding, using the same assay. As evident from the data in Table 1, L-cyst spontaneously bound CDNB independently of the presence GST, at a rate comparable to GSH in the presence of GST.

**Table 1:** Rates of conjugation of 2 mM GSH, NAC or L-cyst with 1 mM CDNB in the presence and absence of 1 μg GST.

**Mix (substrate1,**	**Conjugation Rate**
**substrate 2±enzyme)**	**CR (μM/min)**
GSH+CDNB	1.4±0.3
GSH+CDNB**+GST**	7.0±0.9
GSH+CDNB**+**HYP**+GST**	3.6±0.7
NAC+CDNB	0.4±0.1
NAC+CDNB**+GST**	0.4±0.1
NAC+CDNB**+GST**+GSH	8.0±1.2
L-cyst+CDNB	8.1±1.5
L-cyst+CDNB+**GST**	7.3±0.8

## 4 Discussion

In the present work, the phenotypically diverse breast cancer cell lines MCF7 and MDA-MB-231 expressed different sensitivity to HYP PDT, with MDA-MB-231 cells being approx. 1,7-fold more sensitive than the MCF7 cells. In both cell lines GSH was found to influence their sensitivity to HYP PDT. The cells were however found to utilize the protective effect of GSH towards oxidative stress differently. The MCF7 cells utilized the GSH peroxidase and reductase pathway for protection against ROS formation from HYP PDT while the MDA-MB-231 cells employed glutathione-S-transferase to expel xenobiotics and oxidation products. These mechanistic differences determine the cell sensitivity to HYP PDT and to adjuvant GSH modulating treatments.

Various research groups have previously reported increased PDT-induced cytotoxicity following GSH depletion [18], [19] attributed to GPX-based detoxification. Girotti and co-workers found that transfection of GPX4 into breast cancer carcinoma cell line COH-BR1 not expressing GPX4, made the cells substantially more resistant to PDT (21), indicating the importance of GPX4 in the GSH-based defence against PDT. BSO, an inhibitor of glutathione synthetase, is generally used to evaluate the impact of GSH on treatment efficacy. Indeed, overnight treatment with 100 μM BSO reduced the GSH content by 80% in both cell lines. The BSO pre-treatment had however no effect on HYP PDT sensitivity in the MCF7 cells despite the profound GSH reduction, probably due to their strong GPX4 expression. It was only by an additional inhibition of the glutathione reductase and hence of the intracellular *de salvo* GSH production blocking of by BCNU that an enhanced (differential) cell death was observed. Thus, the intracellular content of GSH appeared much higher than necessary to fulfil the requirement to ensure GPX-based protection against oxidative stress and only by an almost complete removal of GSH the importance of GSH as a protection mechanism could be observed. This is in accordance with our previous work on HYP PDT in DU145 human prostate adenocarcinoma cells [20] as well as other reports.

In contrast to the effects of BSO on MCF7 cell sensitivity to HYP PDT, the suboptimal BSO-induced reduction of intracellular GSH expression was sufficient to enhance the sensitivity of MDA-MB-231 cells to HYP PDT. MDA-MB-231 cells were found not to express GPX4, but strongly express GSTP1 in contrast to MCF7s. GSTs are multifunctional isoenzymes that can detoxify xenobiotics and endogenous metabolites by catalyzing their conjugation to GSH [21]. GSTP1 is overexpressed in tumors and tumor cells resistant to several anticancer drugs [21]. It is important in expelling xenobiotics as well as in the management of lipid oxidation and S-glutathiolated proteins generated by oxidative stress [22]. GSTP1 has also recently been shown to act in conjunction with peroxiredoxin VI, a dual-functioning antioxidant enzyme [23]. GSTP1 has been considered as a marker for cancer development, disease progression as well as drug resistance. The higher sensitivity of the GSTP1 expressing MDA-MB-231 cells to HYP PDT than the GPX4 expressing MCF7 cells and the higher sensitivity to GSH attenuating treatment indicate HYP PDT and GSH depletion adjuvance as attractive treatment options for drug resistant tumors.

N-acetyl cysteine (NAC) is used to load cells with L-cysteine and stimulate an increased synthesis of GSH, mainly by L-cysteine provision through hydrolysis. The 4 h, 3 mM NAC treatment used in the present study induced an approx. 20% increase in GSH expression in the MCF7 cells, but not in the MDA-MB-231 cells. Accordingly, the NAC treatment enhanced the viability of the MCF7 cells, but not the MDA-MB-231 cells after HYP PDT. Surprisingly, when NAC was combined with BSO in the MCF7 cells the GSH content was only marginally increased from that in BSO treated cells, but the viability was as high as in cells treated with HYP-PDT after adjuvant NAC treatment. Taking only the GSH level into account the cells should be almost equally sensitive to those treated with only BSO prior to HYP PDT. However, L-cysteine has been reported to act as a substrate for GPX, although at a slower rate, and may explain the increased viability in NAC-BSO treated MCF7 cells subjected to HYP PDT. A similar effect was not seen of the NAC – BSO combination with HYP PDT on the MDA-MB-231 cells. NAC had no effect on the GSH content of BSO-treated MDA-MB-231 cells as in untreated cells. It cannot be excluded that the loading of the MDA-MB-231 cells with L-cysteine is lower than in the MCF7 cells, but more likely the lack of GPX to utilize cysteine as a substitute for GSH may explain the unchanged sensitivity to HYP-PDT.

GSTs are known to detoxify xenobiotics by conjugating them to GSH to form GS-X complexes [24]. GSH-complexed toxic substances are consequently expelled from the cell via an export carrier, the GS-X pump [16], aka “multispecific organic anion transporter” (MOAT, Fig. 3) [25]. GST-induced GSH binding could thus be the prevalent mechanism of conjugation and consequent expulsion of HYP in the MDA-MB-231 cells. When cells however were treated first with BSO and then with NAC, intracellular GSH was severely depleted (Fig. 4), and excess L-cysteine from NAC hydrolysis could not be used to produce GSH since BSO inhibited glutathione synthetase. Thus, the amount of HYP bound to the MDA-MB-231 cells, but not to the MCF7 cells, should be expected to increase in the presence of both BSO and NAC. It was however found that the BSO-NAC combination reduced the amount of intracellular HYP fluorescence in the MDA-MB-231 cells by 30% compared to cells treated with HYP only (Fig. 6). A similar reduction (¬20%) could be also seen for MCF7 cells which lacked GSTP1. The reduced overall HYP loading following co-treatment with additional supply of L-cysteine by NAC therefore indicated that L-cysteine could contribute to the enhanced excretion of HYP. Interestingly, L-cysteine was highly efficient in non-enzymatic conjugation to the GST substrate CDNB while GSH was highly dependent on the presence of GST (Table 1). One may therefore hypothesize that the amount of available L-cysteine for conjugation to HYP is low in the presence of functional GSH synthetase (in the absence of BSO) and high when GSH synthetase is inhibited. Thus, in the presence of high levels of L-cysteine (cells treated with BSO and NAC) HYP may be non-enzymatically conjugated to L-cysteine and expelled by the GSX pump (Fig. 3). The lower effect of the BSO-NAC combination on the MCF7 cells may be due to a lower activity of the GSX pump which is apparently not needed in cells lacking GST. The slightly increased HYP loading by BSO in MDA-MB-231 cells is in accordance with GSTP1-based excretion of HYP conjugated to GSH.

The study of the importance of the various forms of L-cysteine supply for GST activity showed that HYP (60 μM) could reduce the GST activity (Table 1). This indicates that HYP may be strongly bound to GST, as was also shown elsewhere [26]. Interestingly, in that same work [26a], it was shown that the strong binding of HYP with GSTP1 (K_D_=0.51 μM), suppresses singlet oxygen production to almost negligible levels. In this sense, GSTP1 would protect MDA-MB-231 cells against HYP-PDT, probably both by conjugating HYP to available GSH and binding of HYP to GSTP1.

The expression of glutathione-S transferase (GSTP1) was found to be very strong in MDA-MB-231 cells while hardly detectable in the MCF7 cells. Conversely, MCF7 cells profoundly expressed the membranic glutathione peroxidase (GPX4), which is practically absent in MDA-MB-231. This differentiated use of GSH in the two cell lines can be attributed to the different metabolic phenotype of the two cell lines:

MCF7 cells perform respiratory ATP production at normoxic conditions and also switch to glycolysis under hypoxia, whilst MDA-MB-231 cells rely on glycolysis for ATP production in both normoxic and hypoxic circumstances [7a,b]. Indeed, as verified by our metabolic studies [7], [13], in normal conditions the oxygen consumption rate of MCF7 cells is many fold higher than that of MDA-MB-231 cells. In this context, Pasteur type MCF7 cells, due to their respiratory nature, require more antioxidant protection from electron transport chain leakages and the inevitable creation of free radicals and reactive oxygen species (ROS) such as O_2_^-•^, H_2_O_2_ and deleterious hydroxyl radicals, forming from Fenton reactions catalyzed by transition metals and causing lipid peroxidation [27]. This is in accordance with an upregulation of GPX to protect against radical formation. Warburg type MDA-MB-231 cells, on the other hand, do not require the same level of antioxidant protection since their respiratory activity is profoundly suppressed, and their main use for GSH appears to be to expel harmful xenobiotics and from the cell interior through the GS-X pump (Fig. 3).

In accordance to the above, GPX enzymes and in particular membranic GPX enzymes like GPX-4 can be used as predictive marker of the cell response to PDT and likewise GST enzymes can be used as predictive markers for the chemoresistance of a cell line. MDA-MB-231 cells lacking GPX4 was found more sensitive to HYP PDT than the MCF7 cells and has previously been reported to be most sensitive to TPCS_2a_-PDT of 4 breast cancer cell lines [4a] . In contrast, MDA-MB-231 cells are quite vulnerable to PDT but not to chemotherapy, while MCF7 cells are quite resistant to PDT. The results of this study shown that the glutathione related family of enzymes may be utilized to predict treatment response, select patients for PDT and chemotherapy and choice of GSH-related adjuvant therapies.
